# Supplementary material for: Protein Expression Correlates Linearly with mRNA Dose over Up to Five Orders of Magnitude In Vitro and In Vivo
Source: Biomedicines. 2021 May 5;9(5):511. doi: 10.3390/biomedicines9050511 (PMC8148180; doi:10.3390/biomedicines9050511)
Supplement: Supplementary file 1 [file biomedicines-09-00511-s001.zip › biomedicines-1167635-supplementary.pdf]

Supplementary Materials for

Protein expression correlates linearly with mRNA dose over up to five orders of magnitude in vitro and in vivo

Alexander H. van Asbeck [1,†], Jürgen Dieker [1, †], Rik Oude Egberink [1], Lennard van den Berg [1], Johan van der Vlag [2], Roland Brock [1,3*]

[1] Department of Biochemistry, Radboud Institute for Molecular Life Sciences (RIMLS), Radboud university medical center, 6525 GA, Nijmegen, The Netherlands; vanasbeck@ribopro.eu; dieker@mercurna.com; rik.oudeegberink@radboudumc.nl; lennard.vandenberg@radboudumc.nl;

[2] Department of Nephrology, Radboud Institute for Molecular Life Sciences (RIMLS), Radboud university medical center, 6525 GA, Nijmegen, The Netherlands; johan.vandervlag@radboudumc.nl

[3] Department of Medical Biochemistry, College of Medicine and Medical Science, Arabian Gulf University, Manama, Kingdom of Bahrain

* Correspondence: roland.brock@radboudumc.nl

† Present address: RiboPro B. V., Kloosterstraat 9, 5349 AB, Oss, The Netherlands

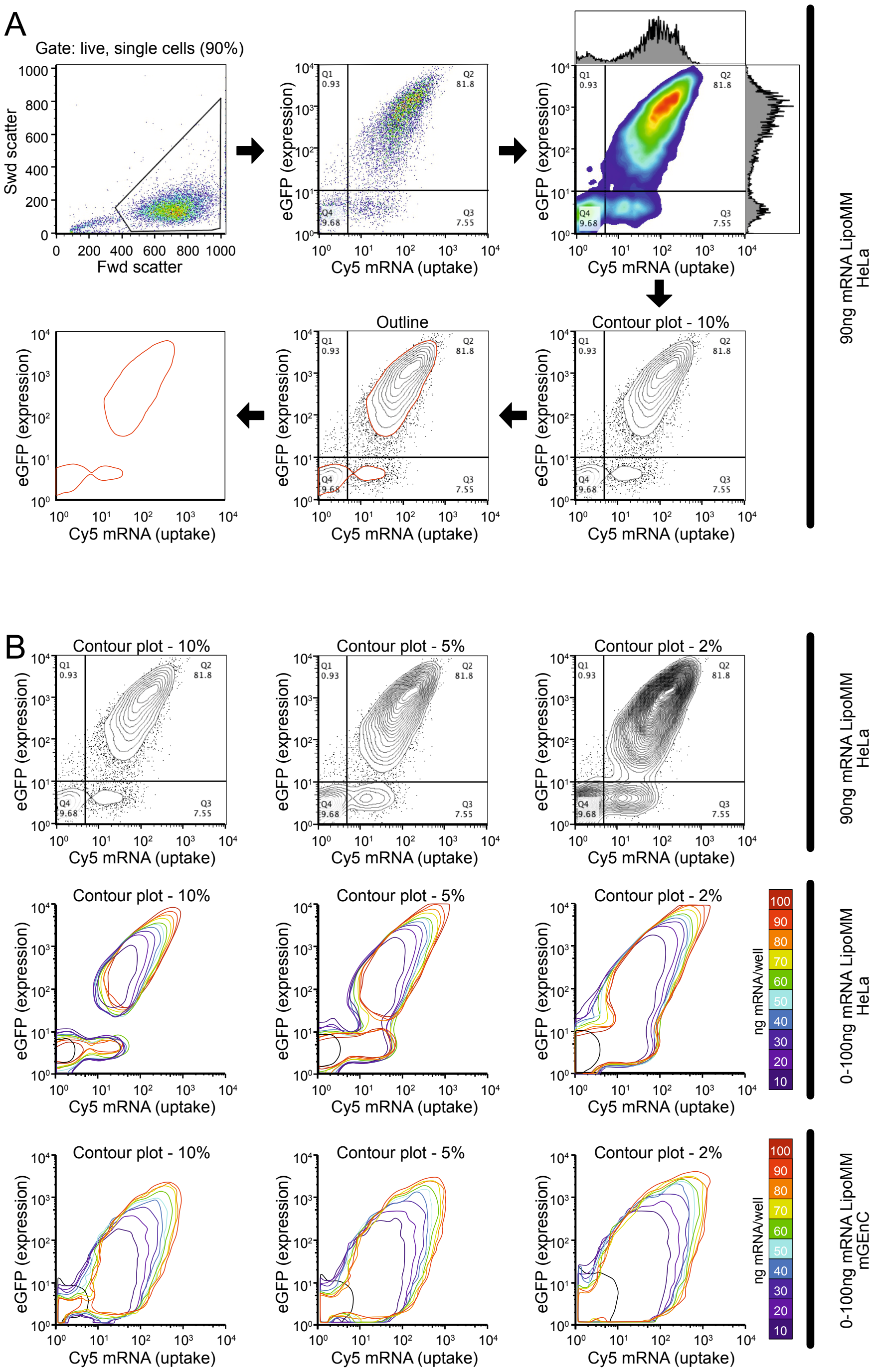

Supplementary Figure S1: (A) Illustration of the gating strategy of viable cells (B) and illustration of the impact of the contour level on the visualization of cell populations. A contour plot at 10 % means that 90 % of events are included in the contour.

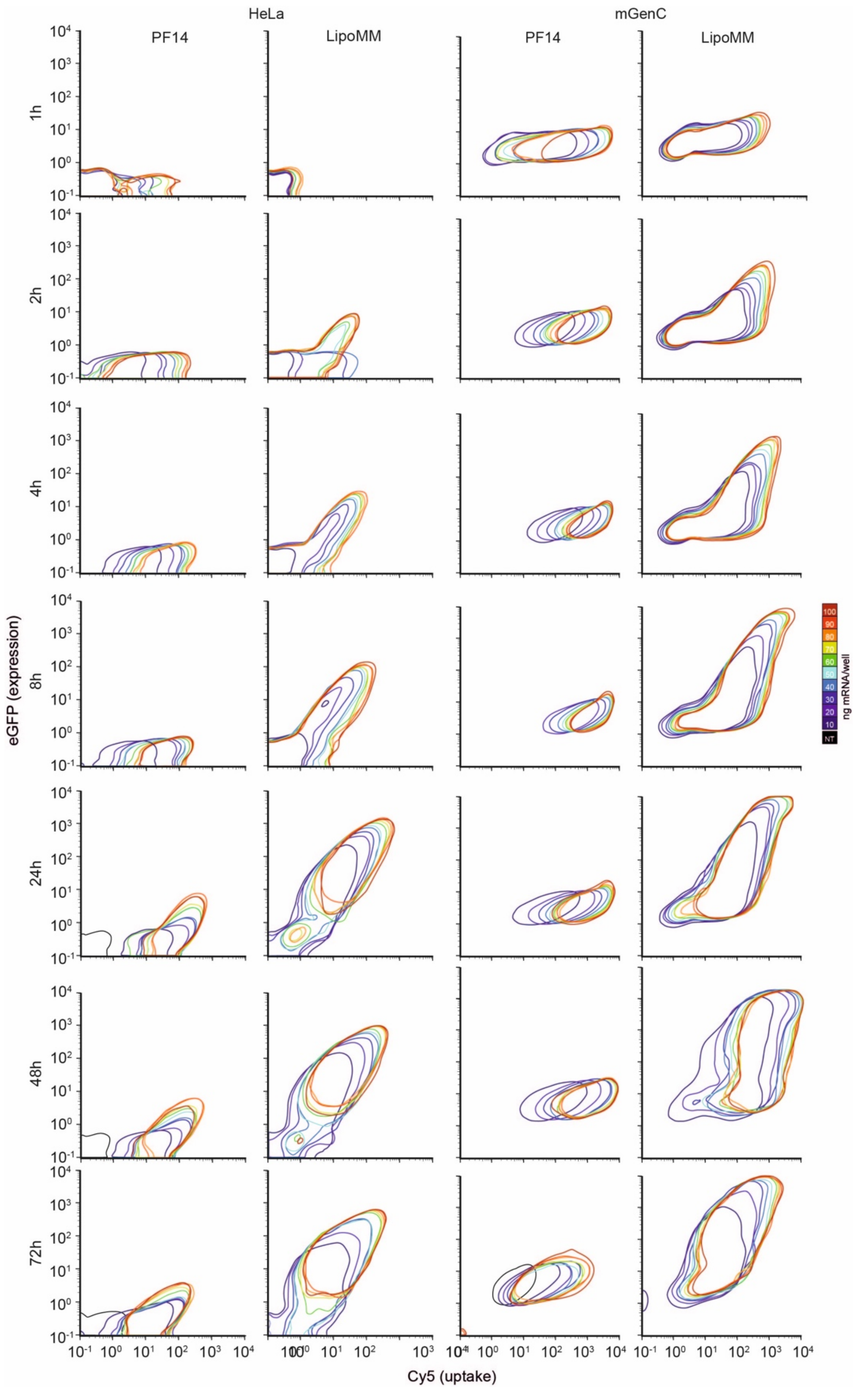

Supplementary figure S2: The entire cell population shows a time-dependent shift of mRNA uptake and protein expression over time. 2D histograms for time dependence of uptake and protein expression for HeLa cells (left panels) and mGEnC (right panels). 5% contour (including 95% of cells) is shown.

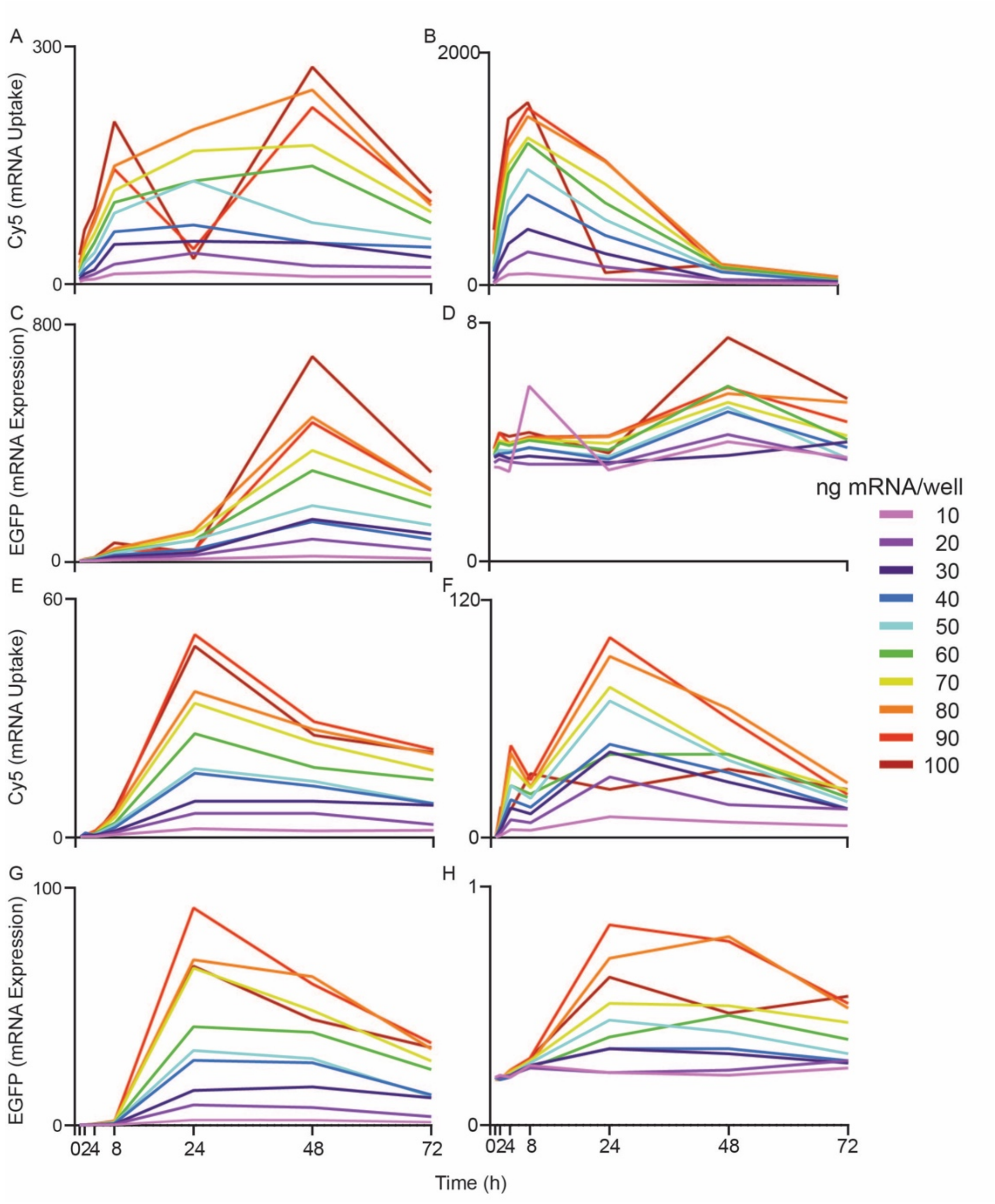

Supplementary Figure S3: The temporal evolution of mRNA uptake and protein expression is largely independent of mRNA dose. (A-D) Time courses of mRNA uptake and (E-H) EGFP expression for mGEnC and HeLa cells. Left columns depict cells treated with LipoMM-formulated mRNA, right columns cells treated with PF14-formulated mRNA. The figure shows the same data as figure 4 with the exception of linear Y-axes scales.

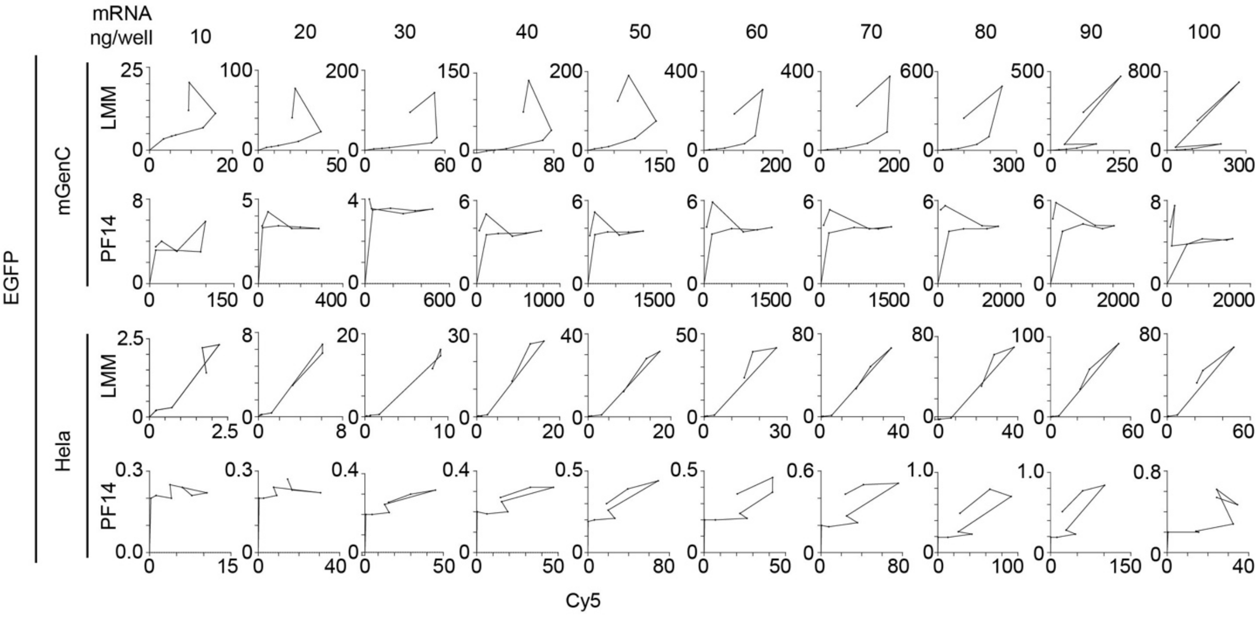

Supplementary Figure S4. The temporal evolution of mRNA uptake and reporter gene expression is a function of cell type and delivery vehicle but independent of mRNA dose. Concentration dependence of the time course of uptake and expression for HeLa cells. Top row, LipoMM, bottom row, PF14. Each panel represents one concentration, and consecutive time points of 1, 2, 4, 8, 24, 48, and 72 h are connected by lines starting from the origin.

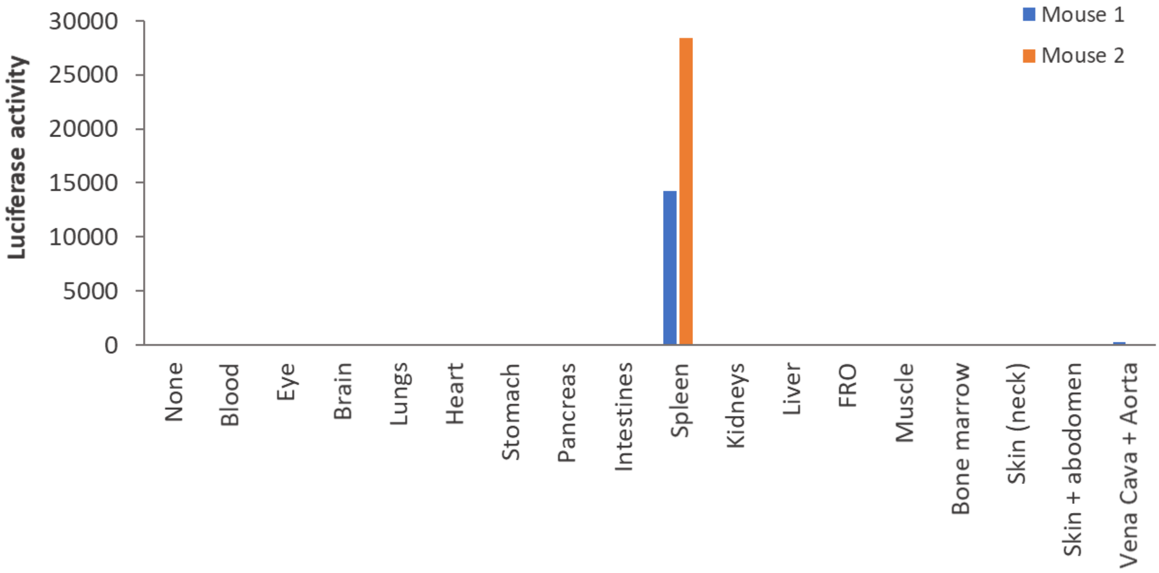

Supplementary Figure S5. Expression of LipoMM-formulated mRNA is restricted to the spleen. Twenty four hours after intravenous injection of 500 µg LipoMM-formulated firefly luciferase reporter gene expression was measured in the indicated organs.

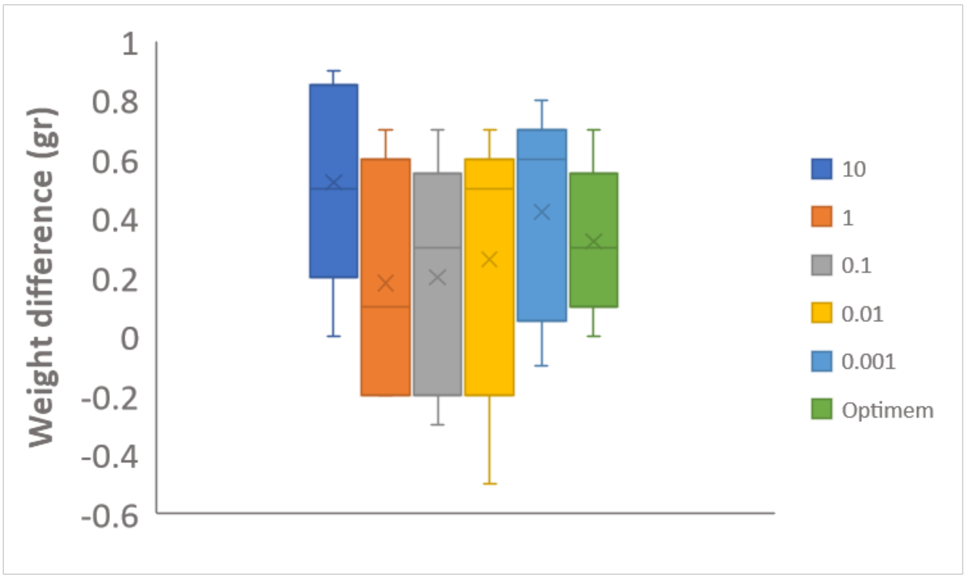

Supplementary Figure S6. The injected doses of mRNA formulation have no impact on animal weight. Weight difference before and 24 hours after injection of the indicated doses of LipoMM-formulated Firefly luciferase mRNA. Shown are averages of five mice per condition.
